# Derivation of human toxicokinetic parameters and internal threshold of toxicological concern for tenuazonic acid through a human intervention trial and hierarchical Bayesian population modeling

**DOI:** 10.1038/s41370-025-00746-6

**Published:** 2025-01-24

**Authors:** Lia Visintin, En-Hsuan Lu, Hsing-Chieh Lin, Yasmine Bader, Truong Nhat Nguyen, Thanos Mouchtaris Michailidis, Sarah De Saeger, Weihsueh A. Chiu, Marthe De Boevre

**Affiliations:** 1 Centre of Excellence in Mycotoxicology and Public Health, Faculty of Pharmaceutical Sciences, Ghent University, Ghent, Belgium; 2 Department of Veterinary Physiology and Pharmacology, Interdisciplinary Faculty of Toxicology, Texas A&M University, College Station, TX USA; 3 Department of Biotechnology and Food Technology, University of Johannesburg, Gauteng, South Africa

**Keywords:** Tenuazonic acid, risk screening, toxicokinetic, iTTC

## Abstract

**Background:**

Tenuazonic acid (TeA), a mycotoxin produced by *Alternaria alternata*, contaminates various food commodities and is known to cause acute and chronic health effects. However, the lack of human toxicokinetic (TK) data and the reliance on external exposure estimates have stalled a comprehensive risk assessment for TeA.

**Objective:**

To bridge this gap, a human TK trial and population-based TK (PopTK) modeling were applied to determine human TK parameters of TeA, and the results were applied for risk screening using population biomonitoring data and threshold of toxicological concern (TTC)-based approaches.

**Methods:**

Ten healthy volunteers participated in the TK trial during which the volunteers ingested a bolus dose of TeA at the (external) TTC (1500 ng/kg bw). Blood, urine, and fecal samples were collected over 48 h and analyzed using UPLC-MS/MS. Concentration-time profiles were fit with a multi-compartmental PopTK model using a hierarchical Bayesian population structure. Utilizing a probabilistic framework, fitted TK parameters were used to derive internal TTC (iTTC) values for comparison to blood and urine biomonitoring data. Risk screening with data from five diverse biomonitoring cohorts was performed using Hazard Quotient (HQ) and probabilistic individual margin of exposure (IMOE) approaches.

**Results:**

TeA was estimated to have a population median half-life of 1.9 [90% CI: 1.4–2.7] hours and volume of distribution of 4.4 [3.1–6.1] L/kg, with inter-individual variability geometric standard deviations of 2.4- and 1.7-fold, respectively. Probabilistic lower confidence bound iTTCs were derived of 0.5 nmol/L in blood and 2.53 nmol/kg-d urinary excretion. Risk screening HQs were mostly >1 for the three blood biomonitoring cohorts and < 1 for the two urinary biomonitoring cohorts; results from probabilistic IMOE calculations were qualitatively consistent.

**Significance:**

A comprehensive human TK study was performed for TeA for the first time, demonstrating the importance of integrating TK and population variability for a more comprehensive risk evaluation, particularly for interpreting biomonitoring data. The results for TeA point to the critical need for toxicity data to move beyond TTC-based risk screening.

**Impact:**

- A critical gap in food safety research was addressed studying the toxicokinetics of tenuazonic acid (TeA) in humans and using these data to derive an internal threshold of toxicological concern (iTTC) for comparison to human biomonitoring data. The innovative approach—combining a human intervention trial with population-based toxicokinetic modeling—accounts for inter-individual variability and provides a more comprehensive understanding of population exposure to TeA. The resulting probabilistic iTTC and risk screening methodologies offer improved tools for interpretation of biomonitoring data. These findings have significant implications for food safety regulations and public health protection, potentially influencing future mycotoxin risk assessment strategies.

## Introduction

Mycotoxins are secondary metabolites produced by toxicogenic fungi, such as *Aspergillus*, *Fusarium*, *Alternaria*, and *Penicillium*. Mycotoxins can contaminate food crops posing major health risks [1] and causing significant economic loss [2]. The widespread presence of mycotoxins in global food crops, estimated to affect nearly 80%, is a growing concern, especially with the predicted increase in mycotoxin prevalence due to climate change [3]. Tenuazonic acid (TeA), the primary mycotoxin secreted by Alternaria alternata, has been shown to have acute and chronic toxicity effects on various organisms [4, 5]. Studies have demonstrated TeA’s acute toxicity in rodents (LD_50_ = 81–186 mg/kg bw) and chicken embryos (LD_50_ = 0.55 mg/egg), along with adverse effects such as vomiting and hemorrhages in animal-feeding trials. In vitro models using human intestinal epithelial barrier (Caco-2) and hepatocyte (HepG2) cells have revealed the cytotoxic properties of TeA, which are enhanced when combined with alternariol monomethyl ether (AME) [6].

The European Food Safety Authority (EFSA) has reported that the European population’s dietary exposure to TeA is significantly higher than that of other *Alternaria* toxins [7]. While adults’ estimated exposure levels only exceed the Threshold of Toxicological Concern (TTC) of 1500 ng/kg-d under high exposure scenarios, infants and toddlers face potential health risks with exposures ranging from 37 to 1614 ng/kg-d [7, 8]. Although Bavaria (Germany) imposed a limit for TeA in sorghum/millet-based infant food [9] and the European Commission recently introduced indicative alert levels for *Alternaria* toxins in specific food commodities[10], *Alternaria* toxins are not currently regulated in foods in any country.

The TTC approach is used by regulatory agencies worldwide to estimate exposure levels at which no appreciable human health risk is expected, in the absence of chemical-specific toxicity data [11–15]. The external TTC for TeA of 1500 ng/kg-d was established by EFSA^1^ following the guidance on the application of TTC for substances for which toxicity data are limited^2^. Specifically, the TTC was applied to *Alternaria* toxins, including TeA, because the chemical structure of the substance is known, there are limited chemical-specific toxicity data, and the exposure can be estimated from existent dietary exposure data. The TTC is derived based on the most conservative No Observed Adverse Effect Levels (NOAELs) values for non-genotoxic compounds within Cramer Class III reported in the Munro dataset. The TTC is defined as the 5^th^ percentile of the NOAELs distribution converted to the intake for a 60-kg person following the application of an uncertainty factor of 100. The external TTC is derived from animal data and reflects a conservative threshold applied in risk assessments where limited substance-specific toxicological data are available. Additionally, the concept of an internal TTC (iTTC) has been proposed to address the exposure-route specificity limitation of the TTC approach, allowing for broader application and comparison with aggregate exposure estimates [14, 16]. The iTTC offers advantages in understanding internal exposure relative to external exposure, integrating chemical-specific properties, and being readily more comparable with human biomonitoring (HBM) exposure data [14, 16–18].

Investigation into the metabolism and excretion profile of TeA in animals and humans has been limited so far [19, 20]. One study on rats found that the urinary excretion rate of TeA is as high as 55% [20]. Two independent studies conducted on human subjects reported a urinary excretion rate of 87–93% after consuming TeA-contaminated food (*n* = 2) [19] and of 39 ± 22% after ingestion of a bolus at the TTC level (*n* = 4) [21]. Moreover, the latter study proved that TeA may be subject to phase I and II metabolism in human. Currently, assessments of dietary TeA exposure are based solely on biomonitoring the mycotoxin itself, making a comprehensive risk assessment almost impossible due to the absence of toxicokinetic (TK) parameters and reference levels. Understanding the potential health consequences in humans necessitates the capability of conducting accurate mycotoxin exposure assessments at the individual level. More research is needed to understand the absorption, distribution, metabolization, and excretion (ADME) properties of TeA, specifically regarding its human excretion profile and internal dose. Mycotoxicology research has started to apply human toxicokinetic trials, with the first trial conducted by Vidal et al. [22] on deoxynivalenol (DON). This trial provided insights into the metabolism and excretion of DON, facilitating the estimation of DON intake using urinary biomarkers [22, 23]. The results were further valorized by Lu et al. [24] by combining the in vivo data with Bayesian modeling, HBM data and in vitro population-based toxicodynamic data within the WHO/IPCS probabilistic framework. The elaboration served to advance the quantitative risk characterization by replacing the TDI with the probabilistic equivalent Human Dose corresponding to a critical effect size M and population incidence I (HD_M_^I^). The collection and analysis of blood, urine and feces is pivotal to allow a comprehensive understanding of TeA fate in the body. Therefore, the protocol of Vidal et al. [22] was adapted as a multi-matrix approach and applied for the investigation of the toxicokinetics of TeA. For the blood collection, Volumetric Absorption Micro-Sampling (VAMS) technique with Mitra® tips was chosen to allow the quantification of TeA in a manner that is both sensitive and user-friendly. By reducing the invasiveness of sampling and being suitable for self-collection, the technique allows the autonomous collection of serial blood samples on multiple days [25]. Through a human intervention trial and targeted Ultrahigh Performance Liquid Chromatography Tandem Mass Spectrometry (UPLC-MS/MS) methods, this study aimed to define the human excretion profile of TeA in urine, capillary blood, and feces. The concentration-time profiles were further elaborated to build a hierarchical Bayesian population model (PopTK) and derive toxicokinetic parameters of TeA. Additionally, the results were applied for the probabilistic derivation of the iTTC for TeA in blood and urine, extending work by Arnot et al. 11, 12. Finally, the iTTC was compared to available HBM data for quantitative risk screening in terms of a provisional hazard quotient and probabilistic individual margin of exposure.

## Material and methods

**Fig. 1:**
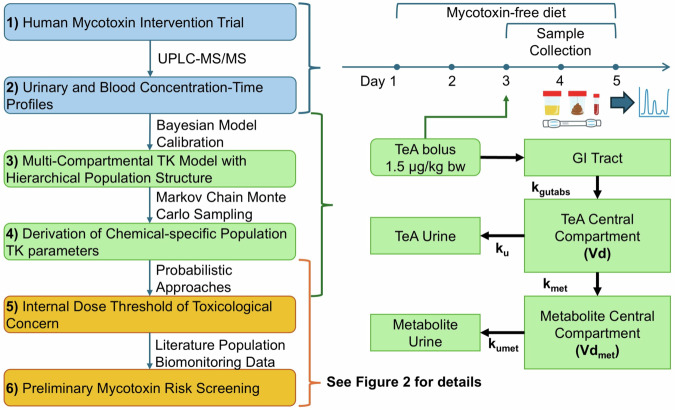
Overview of Materials and Methods, including structure of the multi-compartmental toxicokinetic (TK) model used for TeA. The abbreviations reported represents the gastrointestinal tract (GI tract), volume of distribution of TeA (Vd), volume of distribution of the metabolites (Vd_met_), absorption rate constant (k_gutabs_), urinary excretion rate constant (k_u_), metabolic rate constant (k_met_), and the urinary excretion rate constant of the metabolites (k_umet_).

An overview of the materials and methods is shown in Fig. 1. The study starts with (1) a human mycotoxin intervention trial in which volunteers are given a single bolus dose at the TTC with subsequent collection of blood, urine, and feces, analyzed by UPLC-MS/MS to determine (2) individual concentration-time profiles. These data are fit using (3) a multi-compartment TK model with a hierarchical population structure using MCMC sampling to derive (4) population TK parameters. These are integrated into a probabilistic dose-response/hazard characterization approach adapted from our previous work with DON to derive (5) internal dose-based TTC values for comparison with biomonitoring data in blood and urine. Finally, these are compared with population biomonitoring data from the literature to perform (6) preliminary risk screening. All the codes and files used for PopTK modeling the iTTC derivations are shared via the Github repository https://github.com/liavisintin/TK_TeA.

### Human intervention trial

The invitation to participate in a human intervention study was extended to healthy adults between the age of 20 and 65 years, not pregnant or breastfeeding. Subjects with pathologies involving the liver, gastrointestinal tract, or kidneys, as well as those taking medications that could affect the functionality of these organs, were excluded. The study was conducted on 10 volunteers according to the guidelines laid down in the Declaration of Helsinki and was approved by the Ethical Committee of Ghent University Hospital (UZGent, Gent, Belgium) through an amendment to the original dossier B670201630414. Informed consent was obtained from all volunteers prior to participation, and all the medical aspects of the study were supervised by a medical doctor of the UZGent. The volunteers followed a specific diet developed to minimize the dietary intake of *Alternaria* toxins during the 5-days trial. The diet excluded foods commonly highly contaminated by *Alternaria* toxins [7, 10] such as seeds and derivates, tomatoes and derivates, whole grains, spices, apples, pears among others. On the third day of the trial, and before breakfast, the subjects ingested 5 mL of an aqueous bolus containing TeA at the TTC of 1,500 ng/kg body weight per day, as proposed by EFSA [7]. Urinary and fecal samples were individually collected for 48 h after the bolus intake, with participants recording the void volume for urine and the collection time point of each sample. Blood samples were collected following a time schedule via fingerpick using VAMS Mitra® tips (20 µL). A total of 113 urine, 128 capillary blood, and 32 fecal samples were collected in 48 h. Samples were stored in traditional polypropylene sample containers with a screw cap at −80 °C until the day of the analysis to ensure the stability [26, 27].

### Sample extraction and UPLC-MS/MS analysis

All the samples collected in the framework of the trial were analyzed using previously validated methods published by Visintin et al. [27]. Briefly, the blood samples were exctracted with CH_3_OH from the polymeric Mitra® tips and then divided into two aliquots. One aliquot was subjected to a hydrolysis using β-glucuronidase from *Helix Pomatia*. The fecal samples were freeze dried upon delivery to the laboratory and then extracted with acetonitrile 1% CH_3_COOH. Finally, urine samples were also divided into two aliquots: one part underwent hydrolysis, as with the blood samples, while the second part was extracted using Salt-Assisted Liquid-Liquid Extraction (SALLE). All the five groups of samples obtained were finally analyzed with UPLC-triple quadrupole for the quantification of TeA using a matrix matched calibration and internal standard (IS) compensation.

### Blood-plasma partitioning ratio

Fresh EDTA-whole blood, supplied by Rode Kruis Vlaanderen (Ghent, Belgium), was spiked with TeA analytical standard to reach final concentration of 1 ng/mL for the determination of the blood-plasma partitioning ratio of TeA. Samples were gently vortexed and incubated at 37 °C for 1 h while gently shaking to simulate physiological conditions. The whole blood samples were then divided in two aliquots, one of which underwent centrifugation for 10 min at 4000 × *g* to gather the paired plasma samples. The experiment was performed in triplicate. Before staring the extraction, IS was added to both plasma and whole blood samples. Subsequently, the samples underwent protein precipitation with ice-cold CH_3_CN and were centrifugated for 10 min at 10,000 × *g*. The supernatant was transferred in a new test tube and evaporated. Finally the residue was reconstituted in injection solvent (CH_3_CN/H_2_O/CH_3_COOH, 25/74/1, v/v/v), and analyzed via UPLC-MS/MS accordingly to the method published by Visintin et al. [27]. Samples were quantified using matrix-matched calibration and IS compensation.

### PopTK modeling

The multi-compartmental structure of the PopTK model reported in Fig. 1 was chosen after evaluating the experimental blood and excretion profiles of TeA derived from the human intervention. The model consists of a gastrointestinal (GI) tract, urinary compartments for TeA and its metabolites, and a central compartment for TeA and its phase II forms. It is assumed that 100% of TeA is absorbed from the GI tract into the systemic circulation or central compartment with rate k_gutabs_. From the central compartment, TeA is excreted in urine with rate k_u_, or metabolized with rate k_met_. The metabolites are eliminated from the central compartment in urine with rate k_umet_. The ratio between the amount in the body and the concentration in blood is given by the volume of distribution for TeA (Vd) and its metabolites (Vd_met_). Because urinary TeA measurements includes both free and glucuronidated TeA, and additional parameter F_gluc_ representing the fraction of TeA urinary metabolites that is glucuronidated, and the model fit to the sum of free and glucuronidated TeA. All elimination rates are expressed in hr^−1^ and volume of distribution in L/kg. All parameters were natural log-transformed for fitting.

#### Hierarchical Bayesian population model

A hierarchical Bayesian population model was utilized for TK model fitting and to analyze model parameters, uncertainty, and inter-individual variability [28] as previously described by Lu et al. [24]. The prior distributions of the model parameters were retrieved by allometric scaling from data obtained through a pigs toxicokinetic trial published by Fraeyman et al. [29]. The parameter prior distributions are detailed in Table 1. The TK model fitting was carried out using GNU MCSim v6.1.0 software to perform MCMC simulation and to define posterior parameter values and uncertainty [30]. Four independent MCMC chains of 70,000 iterations each were run. Subsequently, the convergence of the chains was assessed by visual evaluation of the posterior parameters distributions and evaluation of the potential scale reduction factor $\hat{R}$ (≤1.2) [31].

**Table 1:** Human toxicokinetic model parameters, natural logarithm values, and prior distributions set.

Parameter	Description (unit)	Central value	Natural logarithm	Prior distribution for population geometric mean
Cl_tot_	Total clearance of TeA (L/Kg·hr)	0.37	−0.98	LogNormal(0.37, 9.97)
Vd	Volume of distribution of free TeA (L/kg)	0.30	−1.20	LogNormal(0.30, 9.97)
Vd_met_	Volume of distribution of met. phase II TeA (L/kg)	0.30	−1.20	LogNormal(0.30, 9.97)
k_ufrac_	Fraction of free TeA eliminated in urine	NA	NA	TruncLogNormal(0.5, 9.97, 0.01,1)
k_umet_	Phase II metabolites of TeA elimination rate in urine (hr^−1^)	NA	NA	LogNormal(0.5, 9.97)
k_gutabs_	Gut absorption rate (hr-1)	4.96	1.60	LogNormal(4.9, 9.97)
F_gluc_	Fraction of TeA urinary metabolite that is glucuronidated	NA	NA	TruncLogNormal(0.5, 9.97, 0.01,1)

LogNormal(Geometric mean, Geometric standard deviation); TruncLogNormal(Geometric mean, Geometric standard deviation, minimum, maximum).

#### Model fit and predictions for human toxicokinetics

The population and individual TK predictions were evaluated by comparing them with experimental data from the human intervention trial on TeA. The model calibration was checked by correlating predicted and observed experimental values. The distributions of the posterior parameters were evaluated in terms of normality and reliability. The posterior parameter distributions at both population and individual levels were used to generate model predictions of hematic and urinary excretion profiles of TeA and its metabolites. The individual administered TeA mass, body weight, mass of TeA excreted unmodified and modified, and their concentration in blood at each collection time point were input for each subject to simulate the profiles in time. At the population level, parameter values were sampled from the population parameters to reflect uncertainty in their means and variances.

### iTTC derivation

**Fig. 2:**
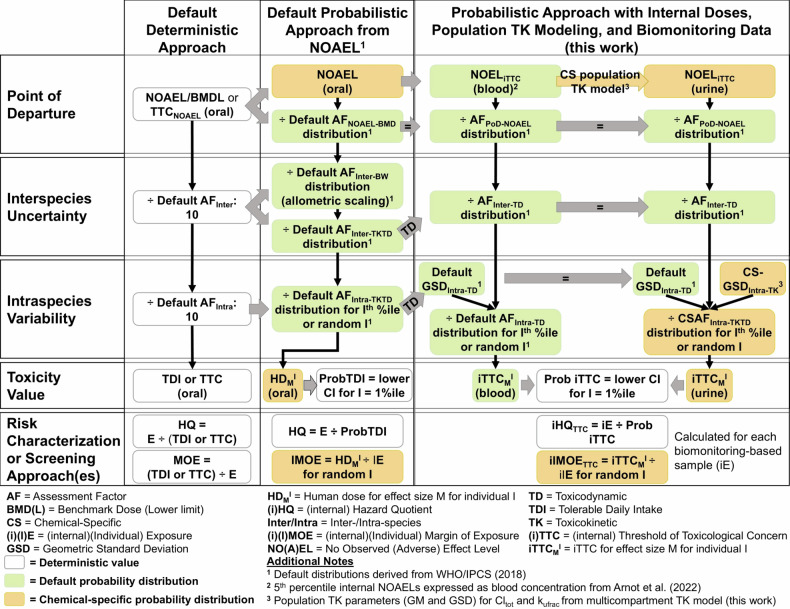
Flowchart illustrating the comparison between the default deterministic approach (left), the default probabilistic approach from No Observed Adverse Effect Levels (NOAEL) (middle), and the probabilistic approach with internal doses, population toxicokinetic (TK) modeling and biomonitoring data (right). The flowchart highlights the differences between the approaches in the steps involved in deriving the internal threshold of toxicological concern and applying risk screening methodologies to tenuazonic acid (TeA).

With no established toxicity value for TeA, we applied the concept of the internal Threshold of Toxicological Concern (iTTC) as an internal exposure benchmark that corresponds more closely to biomonitoring data [11] in blood and urine. Figure 2 shows an overview of the approach, including its relationship with traditional deterministic methods as well as the recommended probabilistic framework for uncertainty and variability from WHO/IPCS [32]. Our derivation followed the similar concept of developing probabilistic biomonitoring equivalents, as described in Lu et al. [24] and consists of 4 components as shown in the following equation:

1 $${{iTTC}}_{M}^{I}=\frac{{{NOEL}}_{{iTTC}}}{{{AF}}_{{PoD}-{NOAEL}}\times {{AF}}_{{interTD}}\times {{AF}}_{{intra}-I}}$$

iTTC_M_^I^ is the internal dose TTC for a critical effect magnitude M and individual I, where I may be a particular population percentile (e.g., I = 1%) or it could represent a random individual (assumed to be drawn from a lognormal variability distribution). The point of departure (PoD) consists of a conservative internal dose NOEL_iTTC_ based on TK modeling by Arnot et al. (2022) of the original Munro et al. dataset used for the TTC. The three assessment factors are (1) AF_PoD-NOAEL_ developed by WHO/IPCS (2018) to address PoD uncertainty for a NOAEL relative to a BMD for M = 5% change in a critical endpoint; (2) AF_inter-TD_ for inter-species toxicodynamic differences (inter-species TK differences are not included because the PoD is already on internal dose basis); and (3) AF_intra-I_ for the human variability factor for individual I. Each component of the iTTC_M_^I^ has an uncertainty distribution, as does the final iTTC_M_^I^. As proposed by WHO/IPCS for external dose toxicity values, a point estimate for the probabilistic iTTC could be defined as the lower 95% confidence bound for I = 1%. Additional details as to each step are described below.

#### Point of departure (PoD)

Our selection of the PoD was sourced from Arnot et al. [11, 12] who compiled external NOEL values from a dataset comprising 613 organic substances with 2941 non-cancer endpoints from oral exposure, based on the original Munro et al. dataset. To translate these external NOELs into internal NOELs, in vivo TK data and in silico TK approaches were applied to convert the values under chemical-specific basis. Three distinct modeling approaches were proposed to calculate steady-state blood concentrations. Each approach assumed different physiological properties to parameterize the model, including variations in first-pass effects and urinary excretion rates. The 5^th^ percentile of cumulative distribution of internal NOEL was selected as the PoD for iTTC. In our study, we incorporated the results of 5^th^ percentile of internal NOEL distributions from the three models used by Arnot et al. [11, 12], and calculated their geometric mean (GM) and geometric standard deviation (GSD). These metrics were randomly sampled to generate the NOEL_iTTC_ uncertainty distribution for TeA. For the urinary iTTC, the internal NOEL uncertainty distribution in blood was converted to 24-hr urinary excretion distribution using the population median estimates of total clearance (Cl_tot_) and free elimination rate in urine (k_ufrac_) from the human population-based TK model described above.

#### AF_PoD-NOAEL_ for point of departure (PoD) uncertainty

WHO/IPCS (2018) [32] noted that PoDs such as the NOEL “may be regarded as a rough estimate of the BMDLx, where x is the default BMR. Thus, the generic uncertainty in the NOAEL may be defined as the precision of the NOAEL in estimating the BMDL.” Therefore, to address the uncertainty arising from the imprecise estimation of the NOEL as the PoD, an adjustment factor AF_PoD-NOAEL_ was applied as recommended. While the original NOEL endpoints collected in Arnot et al. [11, 12] database may encompass both continuous and deterministic endpoints, we opted for the AF_PoD-NOEL_ derived from the continuous type of chronic study [32, 33] as it yielded a more sensitive BMD uncertainty distribution.

#### AF_interTD_ for interspecies factor for TD differences

As the NOEL values underlying the iTTC were derived from experimental animal studies, an interspecies factor needs to be applied to account for interspecies toxicodynamic (TD) differences. Note that interspecies TK differences are not necessary because the PoD is already on an internal dose basis, consistent with the previous case study by Lu et al. [24] for DON. Using the same approach, we note that the default values GM = 1 and GSD = 1.95 from WHO/IPCS [32] were assumed to incorporate equal and independent contributions from TK and TD. Thus, the GSD for TD alone is derived using the formula

2a $${{GSD}}_{{inter\; TK}+{TD}}=\exp \left(\sqrt{\log {\left({{GSD}}_{{interTK}}\right)}^{2}+\log {\left({{GSD}}_{{interTD}}\right)}^{2}}\right)=$$

2b $$\exp \left(\sqrt{2\times \log {\left({{GSD}}_{{interTD}}\right)}^{2}}\right)={{{GSD}}_{{interTD}}}^{\sqrt{2}}$$

The formula leads to the equation ${{GSD}}_{{inter\; TD}}={{{GSD}}_{{inter\; TK}+{TD}}}^{1/\sqrt{2}}$, which was utilized to generate a distribution for interspecies TD differences ${{AF}}_{{interTD}}$.

#### AF_intra-I_ for human variability

WHO/IPCS (2018) [32] assumed independent contributions of TK and TD to human variability. For the probabilistic iTTC in blood, we only estimated human TD variations as human TK variability is inherently considered in steady-state blood concentrations, consistent with the previous case study by Lu et al. [24] for DON. WHO/IPCS (2018) [32] assigned the log scale TD variability in the human equipotent dose distribution ${\log }_{10}\left({{GSD}}_{{intraTD}}\right)$ with P_50_ = 0.221 and P_95_/P_50_ = 2.85, based on observations of human physiological and biological alterations. The ${{GSD}}_{{intraTD}}$ was used to address individual differences of internal dose. We selected incidence I = 1% as the target population percentile, using the corresponding Z-score (power = 2.326) to derive ${{AF}}_{{intra}-I}={{GSD}}_{{intra\; TD}}^{Z}$ for iTTC_M_^I^ in blood. When deriving a value for a random individual, a random Z-score is applied drawn from a standard normal distribution. It should be noted that no TeA data are used in the probabilistic iTTC blood calculation, so the resulting derivation is an applicable to any substance for which the TTC is an appropriate approach.

For the probabilistic iTTC in urine, because blood-to-urine kinetics is chemical-specific, we incorporated the two TK parameters influencing urinary excretion of TeA from our population-based TK model, which are Cl_tot_ (L/kg-hr) and k_ufrac_, to estimate chemical-specific human TK variability. Both the GSD of Cl_tot_ and k_ufrac_ were randomly sampled to generate uncertainty distributions for intraspecies TK as ${{GSD}}_{{intraTK}{{{\rm{\_}}}}{Cltot}}$ and ${{GSD}}_{{intraTK}{{{\rm{\_}}}}{kufrac}}$, respectively. Due to the absence of chemical-specific intraspecies TD data, we adopted the WHO/IPCS (2018) [32] human TD variability uncertainty distribution as previously described. The overall human variability was derived from the assumption of an independent mix of TK and TD, using the formula

3 $$	{{GSD}}_{{intra\; TKTD}}\\ 	=\exp \left(\sqrt{\log {\left({{GSD}}_{{intraTK}{{{\rm{\_}}}}{Cltot}}\right)}^{2}+\log {\left({{GSD}}_{{intraTK}{{{\rm{\_}}}}{kufrac}}\right)}^{2}+\log {\left({{GSD}}_{{intraTD}}\right)}^{2}}\right)$$

As was the case for the blood iTTC, an incidence I = 1% was selected as the target population percentile, using the corresponding Z-score (power=2.326) to derive ${{AF}}_{{intra}-I}={{GSD}}_{{intra\; TKTD}}^{Z}$ for iTTC_M_^I^ in urine, and for a random individual a random Z-score drawn from a standard normal distribution was used.

### Risk screening

#### Human biomonitoring cohorts

The iTTC was further used to perform risk characterisation on data made available from five different cohorts. **Cohort I**—Infant HBM data from the MISAME-III cohort including plasma samples collected during the years 2021–2022 in 6 different rural areas of Bobo-Dioulasso in Burkina Faso [34]. Data on samples collected from 5 infants (aged 11 months) were included in this study. The sample preparation and chemical analysis were according to Vidal et al. [35]. The concentration of TeA was quantified using targeted UPLC-MS/MS analysis. **Cohort II**—HBM data from the University Medical Center Groningen (The Netherlands). Data on plasma samples collected in 2019 from 92 healthy volunteers from The Netherlands were considered in this study. The volunteers were aged 55.5 ± 1.1 years and divided in 49% females and 51% males. The sample preparation and chemical analysis were according to De Ruyck et al. [36]. The concentration of TeA was quantified using targeted UPLC-MS/MS analysis. **Cohort III**—Plasma samples from subjects of the European Prospective Investigation Into Cancer and Nutrition (EPIC) cohort [37], located in ten European countries and aged 35–70 years, were collected between 1992 and 2000 from. In total, 103 HBM data were obtained from the control group via targeted UPLC-MS/MS for precise quantification of multiple-mycotoxin concentration, as described by De Ruyck et al. [36]. The concentration of TeA reported for plasma samples for the three latter cohorts was converted to the concentration in blood using the blood-plasma partitioning ratio determined analyzing fresh spiked human blood and corresponding plasma as detailed above. **Cohort IV**—HBM data obtained from 24 h urinary samples collected by six human volunteers (3 females and 3 males) aged 24–32 years while keeping their individual dietary habits [19]. The sample preparation and chemical analysis were performed according to Asam et al. (2013) [19]. The concentration of TeA was quantified using targeted UPLC-MS/MS analysis post derivatization of TeA. **Cohort V**—HBM data from the European Food Consumption Validation (EFCOVAL) collected from 2006 until 2010 in several European countries [38] and published by De Ruyck et al. [36]. A total of 600 healthy volunteers (aged 45–65 years) were part of this study, including 303 females (50.5%) and 297 males (49.5%). Urine was collected for 24 h from each individual. The biological samples were prepared and analysed according to De Ruyck et al. [36] for TeA quantification by UPLC-MS/MS.

#### Provisional Hazard quotient (HQ) and individual margin of exposure (IMOE)

We assessed the risk level of TeA exposure in three different approaches. First, the “traditional” HQ_TTC_ was determined as the ratio between TeA’s HBM data-derived daily intake estimate and the TTC value. To convert biomonitoring data to estimated daily intake, blood concentrations were initially converted to plasma concentrations using a partitioning ratio of 0.73 ± 0.05, and subsequently calculated to oral doses utilizing the geometric means of TK parameters (Cl_tot_ and k_ufrac_) in our PopTK model. Urinary concentrations were converted to oral doses based on an average 24-h urine volume of 1766.53 mL and a clearance value of 1.56 L/hr-kg bw. The estimated daily intake values were then compared with the oral TTC value for Cramer Class III (1.5 μg/kg bw per day) recommended by EFSA [13]. However, this approach relied on external TTC value to describe exposure, failing to address the effects associated with absorption, distribution, metabolism, and excretion. Moreover, this “traditional” TTC is derived from the deterministic approach without taking into account uncertainty and variability.

To address these limitations, we also applied our derived probabilistic iTTC to evaluate potential risks. We selected the 5^th^ quantile of iTTC_M_^I^ uncertainty distribution as the conservative threshold for comparison with each biomonitoring exposure value. Thus, a provisional HQ_iTTC_ was calculated using the ratio between the biomonitoring exposure value and the 5^th^ quantile of iTTC_M_^I^. However, this HQ_iTTC_ estimation was still based on the conservative assumption from the lower bound of the most sensitive population I = 1%. Lu et al. (2023) [24] previously showed that for DON, this approach may over-estimate population risks.

Thus, to depict the variations in exposure and susceptibility within the entire population, we adapted the “individual margin of exposure (IMOE)” approach [39] for probabilistic risk characterization to this risk screening context. The original IMOE is defined as the ratio between the “Individual Critical Effect Dose” (ICED) and the “Individual Exposure” (IEXP). Both ICED and IEXP reflect both population variability and uncertainty, with the ICED being directly analogous to the HD_M_^I^ as developed by WHO/IPCS (2018) (see Fig. 2). Our adaptation of this approach in this work is as follows:

- We replace IEXP with “internal Individual exposure” (iIE) based on biomonitoring data. To address uncertainty in each biomonitoring cohort, we performed resampling with replacement.
- Because there are inadequate toxicity data for TeA, we replace ICED (i.e., HD_M_^I^) with the probabilistic iTTC_M_^I^ for a random individual.
- To make it clear that we are basing this on a TTC-type PoD, we derive what we denote an “internal, Individual Margin of Exposure for the TTC” (iIMOE_TTC_) as the ratio between the iTTC_M_^I^ and the iIE from biomonitoring data.

As with the original IMOE, uncertainty and variability are separately characterized in a nested loop. First, in the uncertainty (outer) loop, random values are drawn based on the uncertainties in NOEL_iTTC_, AF_PoD-NOAEL_, AF_inter-TD_, and GSD_intraTD_ (for blood) or GSD_intraTKTD_ (for urine), and resampling for iIE. Then, in the variability (inner) loop, random individuals with different z-scores for intra-species variability and different biomonitoring values (iIE) are drawn to derive a distribution of iIMOE_TTC_ = NOEL_iTTC_/(AF_PoD-NOAEL_ x AF_inter-TD_ x GSD_intraTD_^z^ x iIE). An iIMOE_TTC_ below 1 indicates the biomonitoring value exceeds the biomonitoring equivalent iTTC [40]. As an overall summary, at the end of each inner loop, we calculated the probability of iIMOE_TTC_ less than 1 for the population (i.e., fraction of [resampled] individuals with iIMOE_TTC_ < 1). The result after the outer loop is thus a distribution (reflecting uncertainty) of the population at risk of exceeding their individual iTTC.

## Results and discussion

### Blood and excretion profiles

TeA was detected unmodified in urine for 13 h after ingestion, with individual maximum concentrations ranging between 16.11–104.05 ng/mL and 16.94–164.76 ng/mL after hydrolysis. The cumulative urinary excretion of TeA in 48 h accounted for 32 ± 17% of the dose administered. The maximum concentration detected unmodified in capillary blood ranged between 0.11 and 1.87 ng/mL and 0.17–3.13 ng/mL after hydrolysis and was detectable for 6.6 h on average in both cases. All the fecal samples analyzed contained no detectable levels ( < LOD) of TeA. The findings are in line with the results of Puntscher et al. (2019) obtained in rats, where urinary excretion accounted for 17–55% of the administered dose while only 0.6–2% of TeA was eliminated via the GI tract. It was demonstrated that TeA may be involved in both phase I and II metabolism [21]. The use of enzymatic hydrolysis enabled the quantification of phase II metabolites. However, the results did not account for potential oxidative/reductive metabolites, leading to an underestimation of the total metabolite quantities. The multi-compartmental structure was chosen based on these finding, i.e., the elimination via the GI tract was neglected and absorption was assumed to be 100%. The investigation of the partitioning of TeA between whole blood and plasma resulted in a blood-plasma partitioning ratio of 0.73 ± 0.05 (mean ± SD) with a CV% of 6.5%. As there are no regulatory guidelines for acceptable CVs regarding blood-plasma ratios, the threshold was set at 15% as a criterion, based on the EMA criterion for precision in bioanalytical method validation^4^. To the best of our knowledge, this is the first time that blood-plasma ratio for tenuazonic acid is reported.

### PopTK model fit and posterior prediction

**Fig. 3:**
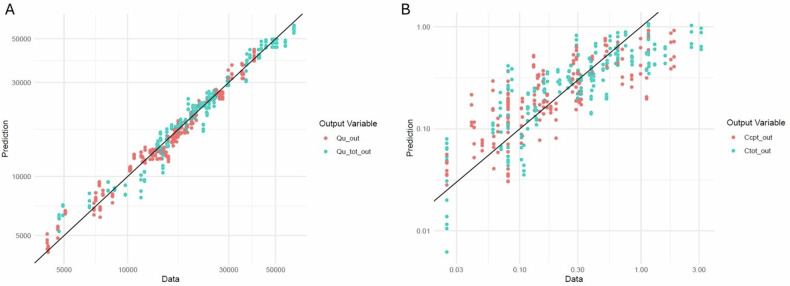
Correlation plots. Correlation plots showing the predicted values versus observed data for the mass of TeA and its phase-II metabolites excreted in urine (**A**) and the blood concentration of TeA (**B**). Qu_out mass of TeA in urine, Qu_tot_out mass of TeA and its phase-II metabolites in urine, Ccpt_out concentration of TeA in blood, Ctot_out concentration of TeA and its phase-II metabolites in blood.

**Fig. 4:**
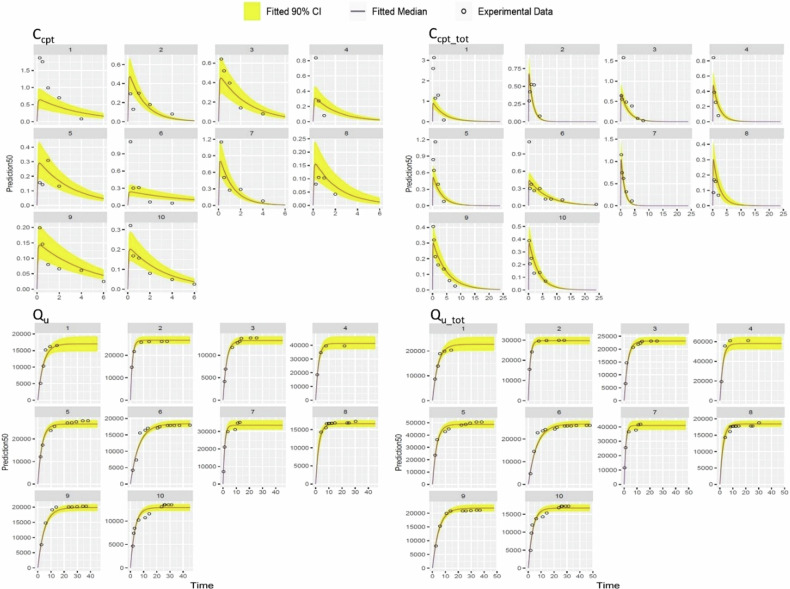
Posterior predictions computed using the population toxicokinetic (TK) model in comparison to the experimental data. The median and 90% confidence interval (CI) are represented by the black line and yellow ribbon, respectively. Each black circle represents an experimental data point. The posterior predictions are divided into four panels, one for each type of experimental data: concentration of tenuazonic acid (TeA) in blood (C_cpt_), concentration of TeA and its phase-II metabolites in blood (C_cpt_tot_), mass of TeA in urine (Q_u_), and mass of TeA and its phase-II metabolites in urine (Q_u_tot_).

The convergence diagnostic (Ȓ) was <1.2 for all population parameters, demonstrating the convergence of the model. The model fit was evaluated through the correlation plot between the predicted and experimental data obtained in urine and blood for TeA across all volunteers (Fig. 3) and individually (Supplementary Material Fig. S1). For both matrices and all matrices, the correlation was linear, indicating a good fit. The cross-correlation plot of each parameter was also evaluated to disprove the hypothesis of (anti-)correlation between parameters, the plots are reported in Supplementary Material Fig. S2. Model’s performance for prediction was further checked by comparing the model prediction and the experimental TK profiles of TeA. Figure 4 shows, for each volunteer, the predictions of the urinary excretion and blood profiles of TeA, along with 90% CI. The model demonstrated a good fit to the data, with nearly all data points falling within the 90% confidence interval, and it is clear that variability between individuals was present.

The prior and posterior distributions of the parameter population means and standard deviations were also compared (Supplementary Fig. S3). The posterior distributions of population means and standards deviations were narrower than the corresponding prior distributions, indicating the informativeness of the data used to model parameters and the population variability. Also in this case, population variability is especially evident in the absorption rate (k_gutabs_) and the clearance of the phase II conjugates (Cl_met_). Table 2 contains the GM and GSD of the population posterior distributions of the TK parameters derived in comparison with the values obtained through allometric scaling of data obtained on pigs published by Fraeyman et al. [29] used to estimate the prior distributions. Allometric scaling from pigs data represented a good starting point for the derivation of the posterior parameters distributions, even though there are clear interspecies differences, e.g., in clearance and volume of distribution values. The Bayesian analysis takes into account the variability in ADME properties among individuals. The results revealed considerable differences between individuals, as can be deducted from Fig. 4, emphasizing the importance of using a population-based model that includes random effects for each parameter to capture this variability effectively. The variability is reflected in the GSD of some of the population posterior distribution such as k_gutabs_. The PopTK model was based on data obtained from 10 volunteers. Therefore, the sample size is a limitation for the representation of the global population. The volunteers were selected to be as representative as possible of the global population. Despite that, the dataset includes a certain level of bias in terms of ethnicity and age group comprising mainly young subjects (80% < 35 years of age; 23–65) and for the 70% Caucasians (20% Hispanic, 10% North African). Regarding BMI and diet, the dataset is more equilibrated covering a wide range of BMI (average 23.0, 18.0–31.8) and diets (plant-based, mediterranean, western European, oriental). Despite these limitations in sample size and demographic representation of global population, the results derived from the model are still valuable. They represent the first attempt to define comprehensive human TK properties of TeA and a significant improvement for HBM and risk assessment as highlighted by the scientific community [41].

**Table 2:** Post-distribution obtained for population model’s and TK parameters of tenuazonic acid (TeA).

Parameter	Unit	Non-compartmental data scaled from pigs		Population posterior distributions Median [90% CI]	
		Average	SD	GM	GSD
k_gutabs_	h^−1^	4.959	12.21	23.9 [12.5–66.2]	1.14 [1.01–1.52]
Cl_tot_	L/(h · kg bw)	0.3748	0.0730	1.58 [1.15–2.04]	1.60 [1.38–1.90]
Cl_met_	L/(h · kg bw)	–	–	33.7 [9.67–286]	1.16 [1.01–1.49]
Vd	L/kg bw	0.3011	0.06858	4.35 [3.10–6.13]	1.67 [1.47–1.94]
Vd_met_	L/kg bw	–	–	7.36 [4.54–13.1]	1.29 [1.04–1.82]
F_met_	–	–	–	0.077 [0.051–0.112]	1.95 [1.68–2.35]
k_ufrac_	–	–	–	0.212 [0.170–0.254]	1.49 [1.35–1.72]
t_1/2_	h	0.5569	2.681	1.94 [1.35–2.74]	2.36 [2.05–2.73]
k_el_	h^−1^	1.245	0.3730	0.357 [0.253–0.512]	2.36 [2.05–2.73]
AUC^a^	ng-h/L	398.74	1.449	952 [774–1310]	1.60 [1.38–1.90]
MAT	h	0.2016	0.08189	0.042 [0.015–0.080]	1.14 [1.01–1.52]
T_max_	h	0.3722	1.394	0.21^b^	1.13^b^
C_max_	ng/ml	4.981	1.061	0.32^b^	1.74^b^

^a^Calculated for a dose of 1500 ng/kg bw.

^b^Calculated from the 10 subjects in the intervention trial.

### Probabilistic iTTC

The results of estimating each element in the derivation of iTTC_M_^I^ are summarized in Table 3 and compared with the corresponding values reported by Arnot et al. [11, 12]. All uncertainty distributions were integrated via Monte Carlo simulation into the equation [1] to derive the iTTC_M_^I^ = 10.3 nmol/L (90% CI: 0.5–241.68 nmol/L) in blood and the iTTC_M_^I^ = 29.95 nmol/kg-d (90% CI: 2.53–263.89 nmol/kg-d) in urine. The lower bound of 90% confidence interval was selected as the probabilistic iTTC. Under the probabilistic approach, a higher probabilistic iTTC value of 0.5 nmol/L in blood was derived compared to the traditional point estimate from Arnot et al. [11, 12] of 0.1 nmol/L in blood, with an approximately 5-fold difference due to the use of probabilistic factors only for TD rather than the full deterministic uncertainty factor of 100. The iTTC was originally introduced to cope with aggregate exposure and route-to-route exposure extrapolation [14]. This parameter is independent by the exposure route and has the advantage that it is directly comparable to HBM data. This makes the iTTC an interesting parameter in the context of risk assessment since it allows to deal with different sources of exposure. In fact, it was proven that the ultimate amount of mycotoxin ingested depends on its bioaccessibility [42]. The bioaccessibility, for its part, is highly dependent on the food matrix, the level of contamination, and possibly the co-presence of multiple mycotoxins [43].

**Table 3:** Results of the derivation of the probabilistic iTTC of TeA.

Component	Arnot et al. [11, 12]	Current study	
	Point estimates	Prob Blood iTTC Median [90% CI]	Prob Urine iTTC Median [90% CI]
**Point of Departure**	Blood C_SS_ = 22/8.5/8.3 nmol/L^a^	NOEL in blood C_SS_ = 11.6 [5.5–24.4] nmol/L^c^	NOEL in 24-hr urinary excretion = 91.0 [39.5–210] nmol/kg-d^i^
**PoD-NOAEL**		AF_PoD-NOAEL_ = 0.333 [0.091–1.567]	AF_PoD-NOAEL_ = 0.333 [0.091–1.567]
**Interspecies Extrapolation**	AF_Inter_ = 10	AF_Inter-TD_ = 1 [0.46–2.16]^d^	AF_Inter-TD_ = 1 [0.46–2.16]^d^
**Intraspecies Variability**	AF_Intra_ = 10		GSD_intraTK_Cltot_ = 1.6 [1.38–1.9]^j^ GSD_intraTK_kufrac_ = 1.49 [1.35–1.72]^j^
		GSD_intraTD_ = 1.66 [0.58–4.68]^e^	GSD_intraTD_ = 1.66 [0.58–4.68]^e^
		AF_IntraTD-I=1%_ = 3.26 [0.28–36.16]^f^	AF_IntraTKTD-I=1%_ = 7.6 [3.8–48.98]^k^
**Internal Toxicity Value**	iTTC = 0.1 nmol/L^b^	iTTC_M_^I=1%^ = 10.3 [0.5–241.68] nmol/L^g^	iTTC_M_^I=1%^ = 29.95 [2.53–263.89] nmol/kg-d^g^
		Prob iTTC = 0.5 nmol/L^h^	Prob iTTC = 2.53 nmol/kg-d^h^

^a^Based on three modeling approaches to convert from NOEL to blood steady-state concentration (C_SS_).

^b^Based on approximating central tendency from three blood iTTC estimates of 0.22, 0.085, and 0.083 nmol/L.

^c^Based on GM and GSD of the three blood C_SS_ estimates.

^d^Assume equal and independent TK and TD in AF_Inter-TK/TD_ from WHO/IPCS (2018) [32].

^e^Assume independent TK and TD in AF_Intra_ from WHO/IPCS (2018) [32].

^f^AF is GSD raised to 2.33 power (z-score for 99%).

^g^M = BMR of 5% change; I = 1% incidence in population.

^h^Prob iTTC is lower (one-tailed) 95% confidence bound on iTTC_M_^I^.

^i^Converted from blood C_SS_ to 24-hr urinary excretion based on population GM for Cltot and kufrac from the population-based TK model in this study.

^j^Based on population GSD for Cltot and kufrac from the population-based TK model in this study.

^k^Assume GSD_intraTKTD_ is independent mix of TK and TD. AF is GSD raised to the 2.33 power (z-score for 99%).

### Risk screening

For each HBM cohort, Table 4 represents a statistical summary of detected biomonitoring concentrations, estimated oral dose, HQ_TTC_, HQ_iTTC_, and the probability of iIMOE_TTC_ below 1. For the three cohorts measuring plasma concentrations, **cohort I** study population revealed a higher exposure, with a median blood concentration of 0.88 ng/mL and a median estimated oral dose of 33.22 μg/kg-d. In contrast, the **cohort III** study population displayed the lowest exposure to TeA, with a median blood concentration of 0.34 ng/mL and a median estimated oral dose of 12.73 μg/kg-d. The **cohort II** study population showed lower exposure to TeA compared to the study of **cohort I**, with a median blood concentration of 0.38 ng/mL and a median estimated oral dose of 14.26 μg/kg-d. Notably, **cohort II** study population exhibited a broader range of exposure, spanning from 0.19 to 12.07 ng/mL. In the two cohorts detecting urinary excretion concentrations, the median urinary concentrations were 0.11 μg/kg-d in **cohort IV** study population and 0.08 μg/kg-d in **cohort V** study population. Despite the similarity in urinary concentrations, we observed a tendency for higher estimated oral doses in **cohort V** population (Median: 0.52 μg/kg-d, ranging from 0.29 to 1.17 μg/kg-d) compared to the study of **cohort IV** (Median: 0.38 μg/kg-d, ranging from 0.02 to 3.19 μg/kg-d). This difference in estimated doses in **cohort V** study may be influenced by the absence of urinary excretion volume data, where assumptions based on 24-h urinary volume estimates from our human TK model were made for unit conversion.

**Table 4:** Detected biomonitoring exposure, estimated dose, and risk screening of exposure to TeA.

Cohort	Matrix	Biomonitoring data^a^	Estimated dose^e^	HQ_TTC_	HQ_iTTC_	Prob iIMOE_TTC_ < 1 ^f^
		Med [25th, 75th] (Min, Max)	Med [25th, 75th] (Min, Max)	Med [25th, 75th] (Min, Max)	Med [25th, 75th] (Min, Max)	Med [25th, 75th] (5th, 95th)
**I – MISAME-III (Burkina-Faso)** ***n*** = **5**	Plasma	0.88 [0.42, 1.91] (0.30, 3.37)^b^	33.22 [16.06, 72.25] (11.35, 127.61)	22.15 [10.70, 48.17] (7.57, 85.07)	8.92 [4.31, 19.41] (3.05, 34.28)	3.6% [0.04%, 18.4%] (0.0%, 46.6%)
**II – Groningen (The Nederlands)** ***n*** = **92**	Plasma	0.38 [0.28, 0.68] (0.19, 12.07)^b^	14.26 [10.66, 25.81] (7.20, 457.58)	9.50 [7.10, 17.21] (4.80, 305.05)	3.83 [2.86, 6.93] (1.93, 122.92)	1.6% [0.6%, 5.4%] (0.0%, 22.2%)
**III – EPIC (Europe)** ***n*** = **103**	Plasma	0.34 [0.27, 0.54] (0.04, 0.99)^b^	12.73 [10.38, 20.35] (1.66, 37.37)	8.49 [6.92, 13.56] (1.11, 24.91)	3.42 [2.79, 5.47] (0.45, 10.04)	0.0% [0.0%, 0.66%] (0.0%, 12.43%)
**IV – Asam et al. (2013) (Germany)** ***n*** = **6**	24h-urine	0.11 [0.09, 0.19] (0.06, 0.25)^c^	0.52 [0.42, 0.89] (0.29, 1.17)	0.35 [0.28, 0.59] (0.19, 0.78)	0.22 [0.18, 0.38] (0.12, 0.50)	0.0 [0.0, 0.0] (0.0, 0.09)
**V – EFCOVAL (Europe)** ***n*** = **40**	Est. 24h-urine	0.08 [0.05, 0.15] ( < LOD, 0.68)^d^	0.38 [0.24, 0.71] (0.02, 3.19)	0.25 [0.16, 0.47] (0.01, 2.13)	0.16 [0.10, 0.30] (0.01, 1.36)	0.0 [0.0, 0.0] (0.0, 0.13)

^a^Unit for blood data: ng/mL, unit for urinary data: μg/kg-d.

^b^The plasma concentration of TeA was converted to blood concentration applying a blood-plasma partitioning ratio of 0.73 ± 0.05.

^c^Cumulative mass excreted in 24 h was converted from µg to μg/kg-d using a fraction of clearance that is urine (population geometric mean of 0.212).

^d^Urinary concentrations were converted from ng/mL to μg/kg-d using an average 24h-urine volume (1766.53 mL) and fraction of clearance that is urine (population geometric mean of 0.212).

^e^Unit for estimated dose: μg/kg-d.

^f^Unit for Prob iIMOE_TTC_ < 1: %.

Additionally, we compared the results between HQ_TTC_ and HQ_iTTC_. In cohorts measuring TeA plasma concentrations, HQ_iTTC_ tended to be nearly 2.5-fold lower than HQ_TTC_. Specifically, the median of HQ_iTTC_ was 8.92 (ranging from 3.05 to 34.28) in the **cohort I** study population, 3.83 (ranging from 1.93 to 122.92) in the **cohort II**, and 3.42 (ranging from 0.45 to 10.04) in the **cohort III**. In the other two cohorts investigating TeA urinary concentrations, HQ_iTTC_ was approximately 1.6-fold lower than HQ_TTC_. It is worth mentioning that the HQ_iTTC_ from the two studies measuring urinary HBM data were nearly close to 0, indicating minimal risks.

Because comparing high-end exposures with conservative TTC or iTTC values may over-estimate the potential risk in the population, we also calculated the probability of having iIMOE_TTC_ < 1 as part of a probabilistic risk screening. In the **cohort I** study population, we observed the highest median probability of iIMOE_TTC_ below 1 at 3.6% with an upper 95% confidence bound of 46.6%. The **cohort II** study population showed a median estimate of 1.6% probability of iIMOE_TTC_ below 1, with upper 95% confidence bound of 22.2%. By contrast, the cohort III study population exhibited lower values, with a median estimate of 0% and upper confidence bound of 12.4%. Moreover, in the two studies focusing on urinary concentrations, even the upper 95% confidence bound for the probability of iIMOE_TTC_ below 1 was nearly zero (~0.1%).

There is a clear difference between the estimated oral doses and risk screening results calculated for cohorts collecting plasma and urine. In fact, the estimated oral doses and consequently HQ_TTC_, HQ_iTTC_, and iIMOE_TTC_ are significantly higher when calculated starting from plasma data. There are several factors that can contribute to this divergence, such as differences between populations included in the cohorts, the type of sample collection, and differences in analytical methods and metabolic pattern. **Cohorts I, II**, and **IV** are specific to relatively small European or African regions, while **cohorts III** and **V** include populations from multiple European countries. Overall, every cohort include subjects with specific food sources with variating contamination levels and different dietary habits that makes a comparison between cohorts difficult. Additionally, **cohort I** includes infants’ data (<11 months) while the other HBM data are relative to adult population. The type of sample collection can also play an important role in the discrepancy between plasma and urine results. The urine data cover a time frame of 24 h for each subject, while the plasma data were obtained via spot sampling introducing uncertainty and making it even more difficult to compare urine and plasma outcomes. Additionally, the plasma samples from **cohort I** and **II** were collected in the morning after fasting while in the samples of **cohort III** no fasting took place. A last uncertainty is represented by the analytical methods used. In fact, data from different matrices and cohorts were analyzed with different UPLC-MS/MS methods by different laboratories. Notably, samples of **cohort I** were analyzed as dried plasma on VAMS® Mitra tips.

## Conclusions

This study constitutes the first endeavor to derive human toxicokinetic parameters for TeA using an intervention trial. The blood and excretion profiles of TeA were determined and fecal excretion was found to be undetectable. A multi-compartmental model developed from the concentration-time profiles of TeA in blood and urine provided novel insights into the ADME of this mycotoxin in humans. The integration of these toxicokinetic parameters within a hierarchical Bayesian population model allowed for the quantification of inter-individual variability, a crucial aspect for a more comprehensive risk assessment. The model demonstrated a good fit to the data, with nearly all data points falling within the 90% confidence interval. The analysis took into account the variability in ADME properties among individuals, revealing considerable differences between subjects and emphasizing the importance of using a population-based model to capture the variability effectively. The results of the model were used to derive probabilistic biomonitoring-equivalent internal thresholds of toxicological concern (iTTCs) for TeA in blood and urine; the probabilistic methodology resulted in a 5-fold difference higher iTTC in blood as compared to the point estimate from Arnot et al. [11, 12] due to use of probabilistic uncertainty factors as well as chemical-specific factors based on TeA ADME.

A risk screening performed on five independent population biomonitoring cohorts highlighted the informativeness of using the iTTC for a more comprehensive risk assessment. For the cohorts measuring TeA in plasma, almost all of the individuals tested had levels above the blood iTTC, while in the cohorts investigating TeA urinary concentrations almost all individuals had levels below the urine iTTC. Because direct comparison between a conservative iTTC and exposure may overestimate potential risk, a probabilistic risk screening adapting the individual margin of exposure approach was also applied. Qualitatively, the results were similar, with a higher probability of iIMOE_TTC_ below 1 for the cohorts measuring plasma concentrations compared to the cohorts focusing on urinary concentrations, where the probability was nearly negligible. However, this probabilistic calculation shows there is very high uncertainty in the more highly exposed cohorts, with the confidence interval for the probability of iIMOE_TTC_ < 1 ranging from 0 to up to nearly 50%.

There are several contributions to this large uncertainty in risk screening. On the exposure side, factors include population’s dietary habits, type of sample collection, and analytical methods used to gather HBM data. Future investigations should focus on understanding the discrepancy in risk screening performed staring from hematic matrices compared to urine possibly collecting matched and serial urine and blood samples. On the hazard side, the risk screening results point to the need to prioritize generation of toxicological data to enable a comprehensive hazard characterization of TeA, so that the screening-level TTC can be replaced by a toxicity value specific to TeA. Other uncertainties, such as additional TK data and especially TD data to better characterize inter- and intraspecies variability, are also important to address, though their contribution is less than the overall lack of information from which to generate PoDs.

Together, this study suggests a stepwise workflow for biomonitoring-based risk screening for substances without health-based guidance values: (1) characterize population TK through a human trial and TK modeling; (2) utilize these results to derive substance-specific iTTC values in biomonitoring matrices such as blood or urine; (3) for substances where initial risk screening indicates HQ_iTTC_ > 1, utilize the probabilistic iIMOE_TTC_ approach to characterize uncertainty and variability in hazard potential; and (4) prioritizing substances with highest potential for iIMOE_TTC_ > 1 for development of guidance values and/or toxicological data generation. In applying this workflow to TeA, this study has demonstrated the development and integration of human population TK, probabilistic iTTC derivation, and probabilistic risk screening to provided valuable insights into the potential health hazards associated with TeA exposure, ultimately pointing to the critical need for toxicity data on TeA to characterize risk.

## Supplementary information

Supplementary information

## Data Availability

The codes and datasets generated and/or analyzed during the current study are available from the corresponding repository, additional information is available from the corresponding author on reasonable request.
