# Supplementary material for: Derivation of human toxicokinetic parameters and internal threshold of toxicological concern for tenuazonic acid through a human intervention trial and hierarchical Bayesian population modeling
Source: J Expo Sci Environ Epidemiol. 2025 Jan 24;35(4):632–43. doi: 10.1038/s41370-025-00746-6 (PMC7617506; doi:10.1038/s41370-025-00746-6)
Supplement: Supplementary file 1 — Supplementary information [file 41370_2025_746_MOESM1_ESM.pdf]

Supplementary Materials

# Derivation of human toxicokinetic parameters and internal threshold of toxicological concern for tenuazonic acid using a human intervention trial and hierarchical Bayesian population modeling

Lia Visintin[1], En-Hsuan Lu[2], Hsing-Chieh Lin[2], Yasmine Bader[1], Truong Nhat Nguyen[1], Thanos Mouchtaris-Michailidis[1], Sarah De Saeger[1,3], Weihsueh A. Chiu[2], Marthe De Boevre[1].

[1] Centre of Excellence in Mycotoxicology and Public Health, Department of Bioanalysis, Faculty of Pharmaceutical Sciences, Ghent University, Ghent, Belgium;

[2] Department of Veterinary Physiology and Pharmacology, Interdisciplinary Faculty of Toxicology, Texas A&M University, College Station, USA.

[3] Department of Biotechnology and Food Technology, University of Johannesburg, Gauteng, South Africa.

**Figure S1**: correlation plot between predicted and experimental blood and urine data for tenuazonic acid (TeA) and its phase-II metabolites for the individual volunteers.

**Figure S2**: cross-correlation plot between TK model's parameters. Total clearance of TeA ($Cl_{tot}$), clearance of TeA's metabolites ($Cl_{met}$), fraction of TeA excreted in urine ($k_{ufrac}$), volume of distribution of TeA ($V_{dist}$), volume of distribution of TeA's metabolites, gut absorption rate ($k_{gutabs}$), fraction of TeA metabolized ($F_{gluc}$).

**Figure S3**: The prior and posterior distributions of the parameter population means and standard deviations. Mean (M), standard deviation (SD), total clearance of TeA ($Cl_{tot}$), clearance of TeA's metabolites ($Cl_{met}$), fraction of TeA excreted in urine ($k_{ufrac}$), volume of distribution of TeA ($V_{dist}$), volume of distribution of TeA's metabolites, gut absorption rate ($k_{gutabs}$), fraction of TeA metabolized ($F_{gluc}$).

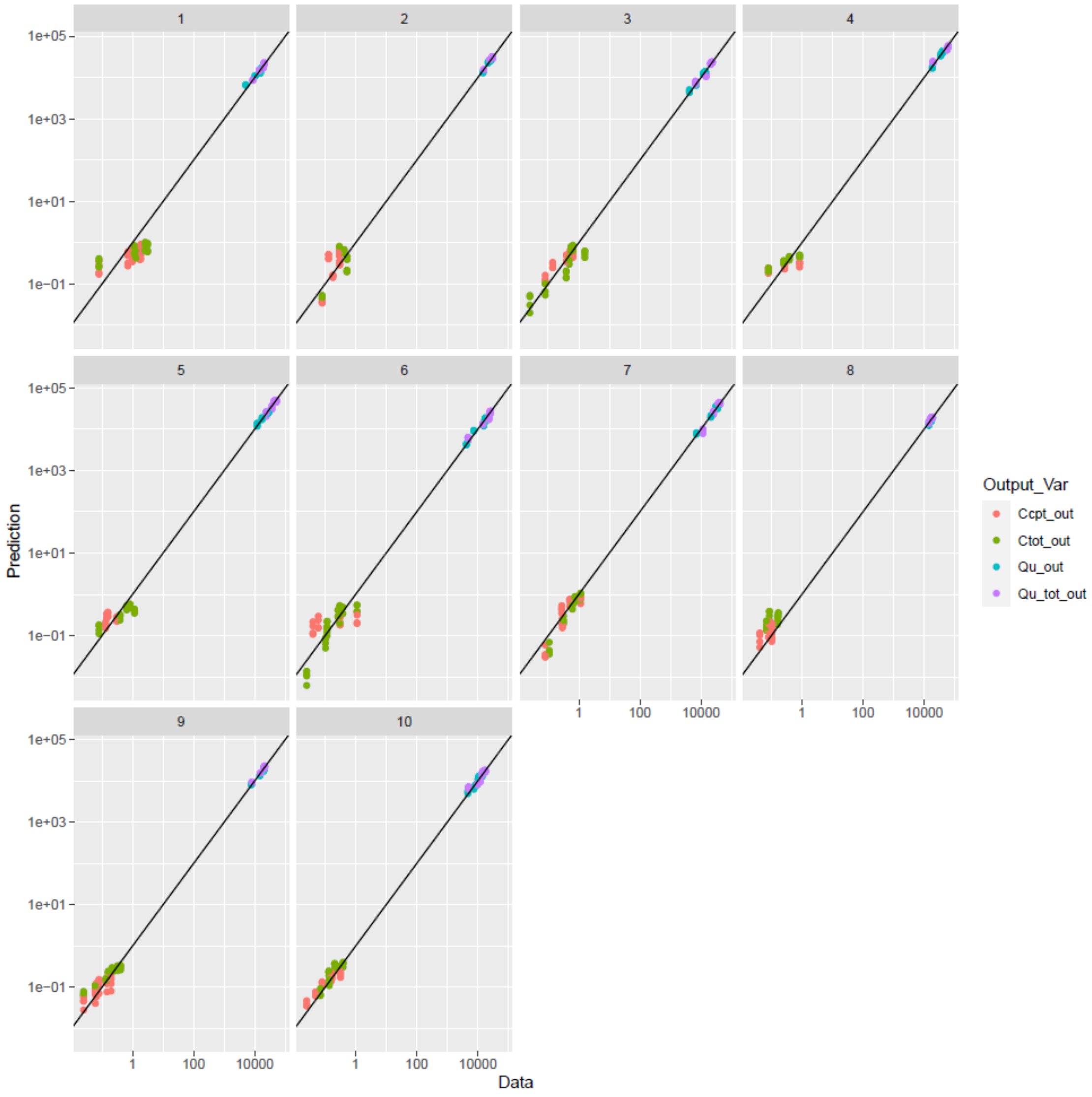


**Figure S1**: correlation plot between predicted and experimental blood and urine data for tenuazonic acid (TeA) and its phase-II metabolites for the individual volunteers.

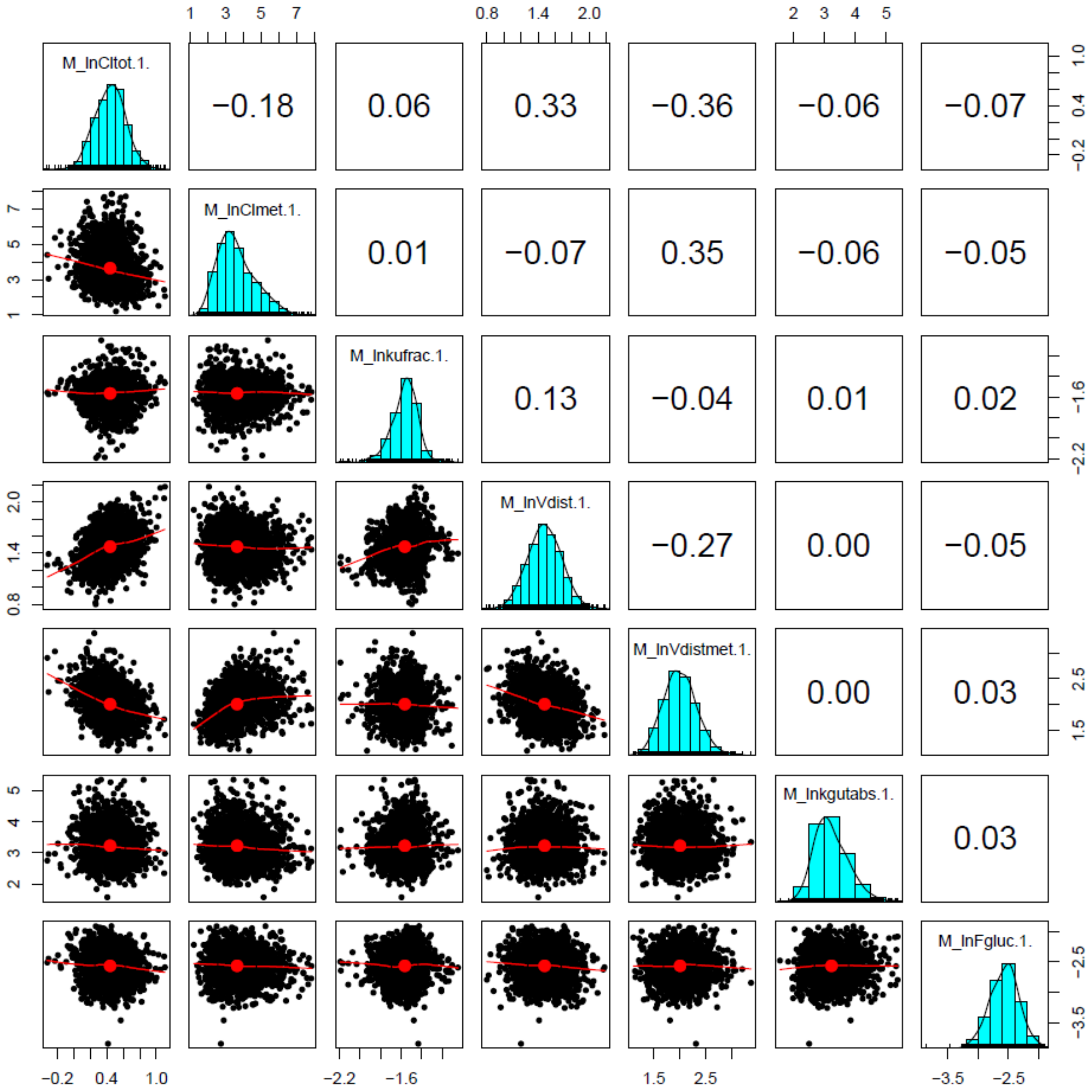


**Figure S2**: cross-correlation plot between TK model's parameters. Total clearance of TeA ($Cl_{tot}$), clearance of TeA's metabolites ($Cl_{met}$), fraction of TeA excreted in urine ($k_{ufrac}$), volume of distribution of TeA ($V_{dist}$), volume of distribution of TeA's metabolites, gut absorption rate ($k_{gutabs}$), fraction of TeA metabolized ($F_{gluc}$).

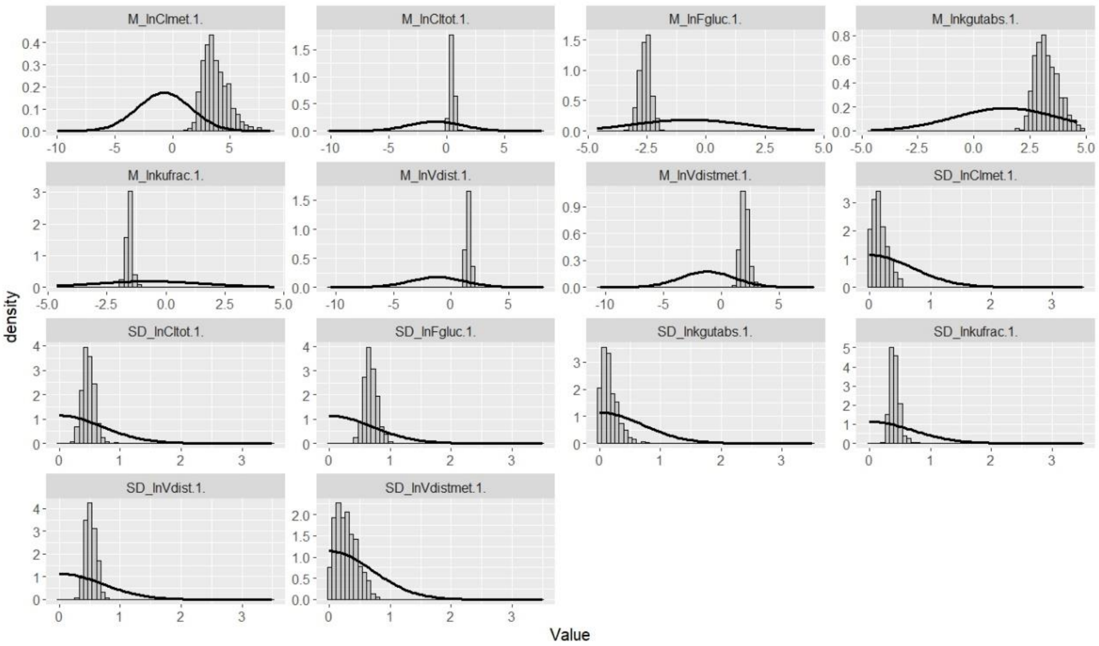


**Figure S3**: The prior and posterior distributions of the parameter population means and standard deviations. Mean (M), standard deviation (SD), total clearance of TeA ($Cl_{tot}$), clearance of TeA's metabolites ($Cl_{met}$), fraction of TeA excreted in urine ($k_{ufrac}$), volume of distribution of TeA ($V_{dist}$), volume of distribution of TeA's metabolites, gut absorption rate ($k_{gutabs}$), fraction of TeA metabolized ($F_{gluc}$).
